# The molecular chaperones DNAJB6 and Hsp70 cooperate to suppress α-synuclein aggregation

**DOI:** 10.1038/s41598-017-08324-z

**Published:** 2017-08-22

**Authors:** Francesco A. Aprile, Emma Källstig, Galina Limorenko, Michele Vendruscolo, David Ron, Christian Hansen

**Affiliations:** 1 Department of Chemistry, University of Cambridge, Cambridge, CB2 1EW UK; 2 Cambridge Institute for Medical Research, University of Cambridge, Cambridge, CB2 0XY UK; 3 Molecular Neurobiology, Department of Experimental Medical Science, BMC B11, 221 84 Lund, Sweden

## Abstract

A major hallmark of Parkinson’s disease (PD) is the presence of Lewy bodies (LBs) in certain neuronal tissues. LBs are protein-rich inclusions, in which α-synuclein (α-syn) is the most abundant protein. Since these inclusions are not present in healthy individuals, despite the high concentration of α-syn in neurons, it is important to investigate whether natural control mechanisms are present to efficiently suppress α-syn aggregation. Here, we demonstrate that a CRISPR/Cas9-mediated knockout (KO) of a DnaJ protein, DNAJB6, in HEK293T cells expressing α-syn, causes a massive increase in α-syn aggregation. Upon DNAJB6 re-introduction into these DNAJB6-KO HEK293T-α-syn cells, aggregation is reduced to the level of the parental cells. We then show that the suppression of α-syn aggregation is dependent on the J-domain of DNAJB6, as the catalytically inactive protein, which carries the H31Q mutation, does not suppress aggregation, when re-introduced into DNAJB6-KO cells. We further demonstrate, that the suppression of α-syn aggregation is dependent on the molecular chaperone Hsp70, which is consistent with the well-known function of J-domains of transferring unfolded and misfolded proteins to Hsp70. These data identify a natural control strategy to suppress α-syn aggregation and suggest potential therapeutic approaches to prevent or treat PD and related disorders.

## Introduction

Parkinson’s disease (PD) is the most common neurodegenerative movement disorder. The cardinal motor symptoms of PD are primarily associated with the selective loss of dopaminergic neurons in the *substantia nigra pars compacta* (*SNpc*) region of the brain^1–4^. This neurodegenerative process correlates with the formation of large protein-rich cytoplasmic inclusions, known as Lewy bodies (LBs), in which aggregated α-synuclein (α-syn) is the main protein component^2–5^.

The *SNCA* gene, which encodes α-syn, is also linked to familial forms of PD caused by gene duplication/triplication and missense mutations that result in increased aggregation of the protein. It is therefore believed that α-syn aggregation plays a key role in PD pathogenesis^1–4^. α-syn is an intrinsically disordered protein of 140 amino acids, which is abundantly expressed in the brain, where it can account for up to 1% of the total protein content of neurons^6^. This protein is found nearly in all neuronal compartments, but it is enriched at the presynaptic terminals, where it has been shown to play a role in vesicular trafficking and neurotransmitter release, in particular by associating with the SNARE complex proteins^7^.

α-syn does not normally form large aggregates in neurons, despite being present in high concentrations, because of its intrinsic solubility and of the presence of an effective protein homeostasis system^8–11^. In particular, one of the main mechanisms to prevent aggregation of α-syn, and more generally of misfolded proteins, is mediated by molecular chaperones. In this context, the 70 kDa heat shock protein (Hsp70) has been reported to have a major protective role against protein aggregation. Specifically, a series of recent *in vitro* and *in vivo* studies have demonstrated that Hsp70 can prevent aggregation of α-syn^11–14^. However, a large body of work has shown that Hsp70 does not normally recruit protein substrates *in vivo*, but does so via an ATP-dependent transfer from other proteins, named co-chaperones, such as the DnaJ/Hsp40 proteins^15^.

There are at least 41 DnaJ proteins encoded in the human genome^15^. Among them, DNAJB6 is expressed in neurons, and has been found to be present in LBs in PD patients^16^. In addition, DNAJB6 is able to inhibit the formation of amyloid aggregates of proteins and peptides, such as Aβ^17^ and polyQ^18–20^, when overexpressed in cell culture. However, all studies so far exploring if DNAJB6 affects aggregation of amyloid proteins, have been based on overexpression studies, and not in more physiologically relevant conditions by making the KO of the protein. Furthermore, no DnaJ protein has so far been shown to inhibit α-syn aggregation in cells, despite the evidence for involvement of Hsp70 in preventing α-syn aggregation^11–14^. Here, we show that DNAJB6 is a suppressor of α-syn aggregation *in cells* by knocking out the expression of endogenous levels of DNAJB6 using the CRISPR/Cas9 system in a stable HEK293T cell line overexpressing α-syn. We then demonstrate that the cooperation between DNAJB6 and Hsp70 is crucial to the inhibition of α-syn aggregation.

## Results

**Figure 1:**
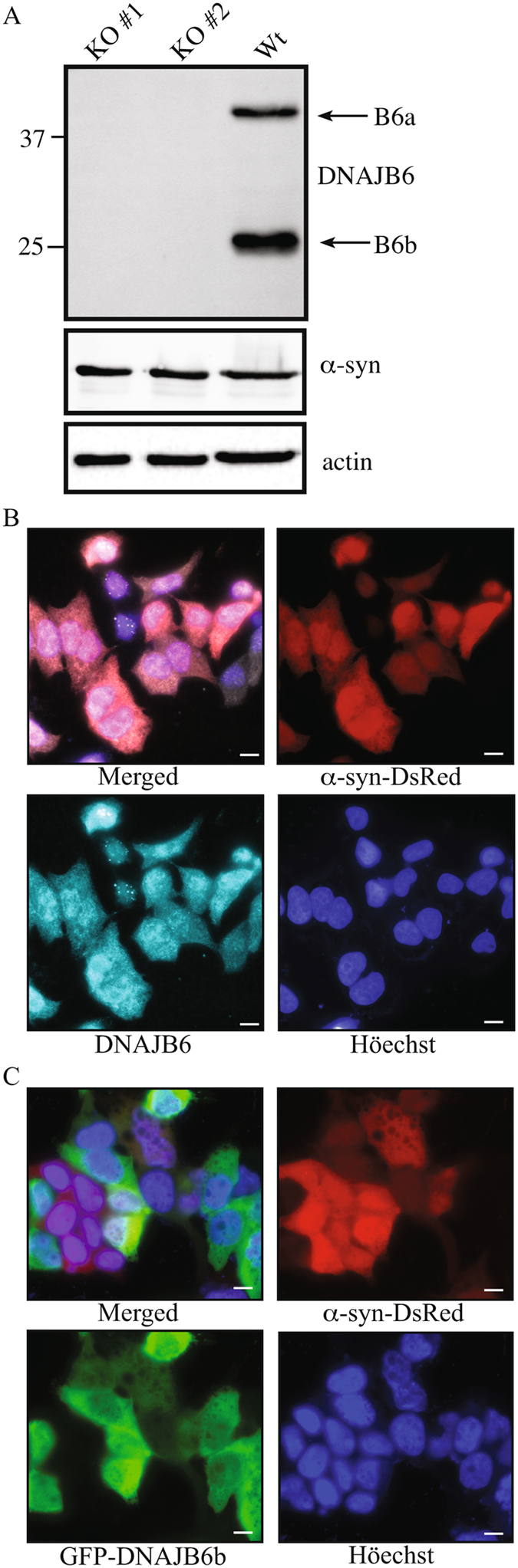
Expression and KO of *DNAJB6* in HEK293T-α-syn-DsRed cells. (**A**) Western blot depicting KO of DNAJB6 expression in α-syn-DsRed HEK293T clone 1 and 2, as analyzed by probing the membrane with anti-DNAJB6 and anti-α-syn antibodies, respectively, as well as RDye fluorescently labeled secondary antibodies. (**B**) Immunocytochemistry showing expression of the endogenous DNAJB6a and DNAJB6b forms in α-syn-DsRed HEK293T cells. Cells were stained with 1^st^ anti-DNAJB6 and 2^nd^ anti-rabbit dylight 649 antibodies. (**c**) Flourescence microscopy displaying expression and localization of GFP-DNAJB6 in transfected HEK293T-α-syn-DsRed cells. Scalebar: 10 μM.

In order to assess if DNAJB6 can act as an endogenous suppressor of α-syn aggregation, we designed RNA guides (gRNA) to specifically target the *DNAJB6* gene and used the CRISPR/Cas9 system^21^ for disruption of endogenous expression of the *DNAJB6* gene in HEK293T cells. For these experiments, we chose HEK293T cells expressing a transgene encoding α-syn fused to the red fluorescent protein DsRed to serve as a sentinel for α-syn aggregation^22^. After transfection with a plasmid encoding both gRNA and GFP-tagged CAS9, single GFP-positive cells were sorted by FACS, clonally-expanded and analyzed genotypically. Of 72 clones tested, 2 were found to have complete knockout of the *DNAJB6* alleles (Fig. 1A).

These clones were found to have the same level of total α-syn as the parental cell line. As seen by the western blot stained for DNAJB6, there are 2 splice forms of the protein resulting in a longer isoform (DNAJB6a) and a shorter one (DNAJB6b) (Fig. 1A). Staining of HEK293T cells expressing the α-syn-DsRed sentinel with anti-DNAJB6 antibody, showed both nuclear and cytoplasmic staining for DNAJB6 (Fig. 1B), whereas transfection with a plasmid encoding GFP-tagged DNAJB6b showed the shorter DNAJB6b isoform is mainly expressed in the cytoplasm (Fig. 1C). Staining of endogenous DNAJB6 in the nucleus of (Fig. 1B) is therefore presumably due to DNAJB6a expression, as DNAJB6a contains a nuclear localization signal. α-syn-DsRed was found both in cytoplasm and nucleus of HEK293 cells and did not overall redistribute in parental (WT) cells relative to KO cells (Supplementary Figure 1).

**Figure 2:**
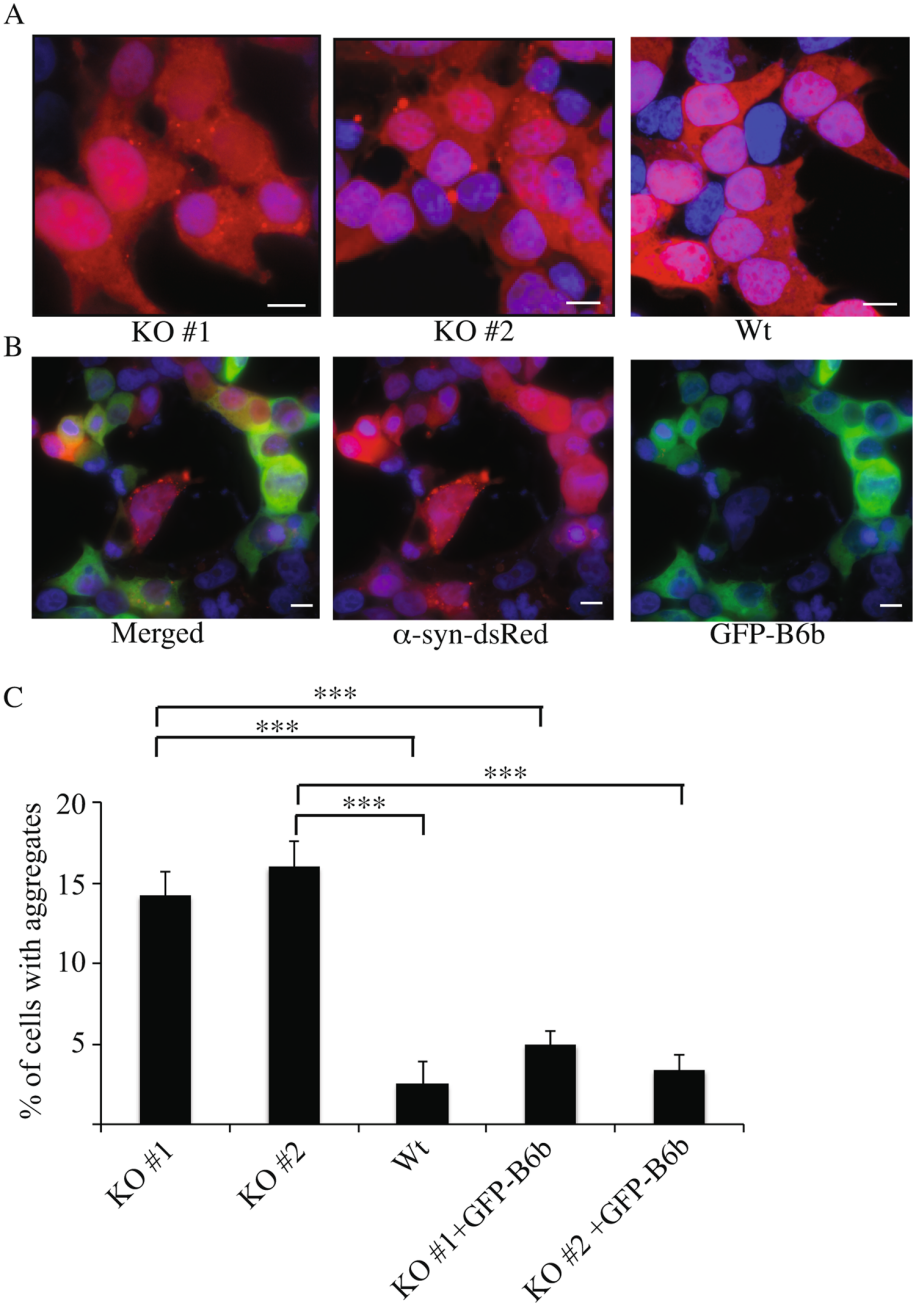
KO of DNAJB6 causes increased aggregation of α-syn in HEK293T-α-syn-DsRed cells. (**A**) Representative pictures showing aggregates (red puncta) in α-syn-DsRed KO clone 1 and 2 relative to parental α-syn-DsRed-HEK293T cells. (**B**) Rescue experiment showing that aggregation is suppressed in KO cells transfected with GFP-DNAJB6b. (**C**) Quantification of aggregation in KO compared to parental cells and KO cells transfected with GFP-DNAJB6 (n = 3). Statistical analysis was performed by one-way ANOVA. ***P < 0.001. Scalebar: 10 μM.

Interestingly however, the DNAJB6 KO clones contained significantly more red puncta (α-syn-DsRed aggregates) than parental cells with a wild type complement of DNAJB6. About 15% of each KO clone harbored puncta compared with about 2% of the parental cells (Fig. 2A,C). To establish the role of the DNAJB6 deficiency in this phenotype, we re-introduced GFP-tagged DNAJB6b into the KO cells. Aggregation in these *trans*-rescued DNAJB6b KO cells was suppressed to the level of the parental cell line (Fig. 2B,C). Elimination of the DNAJB6a isoform only did not cause an increased aggregate formation in α-syn-DsRed expressing HEK293T cells, suggesting that the DNAJB6b isoform is responsible for suppressing α-syn aggregation (Supplementary Figure 2).

**Figure 3:**
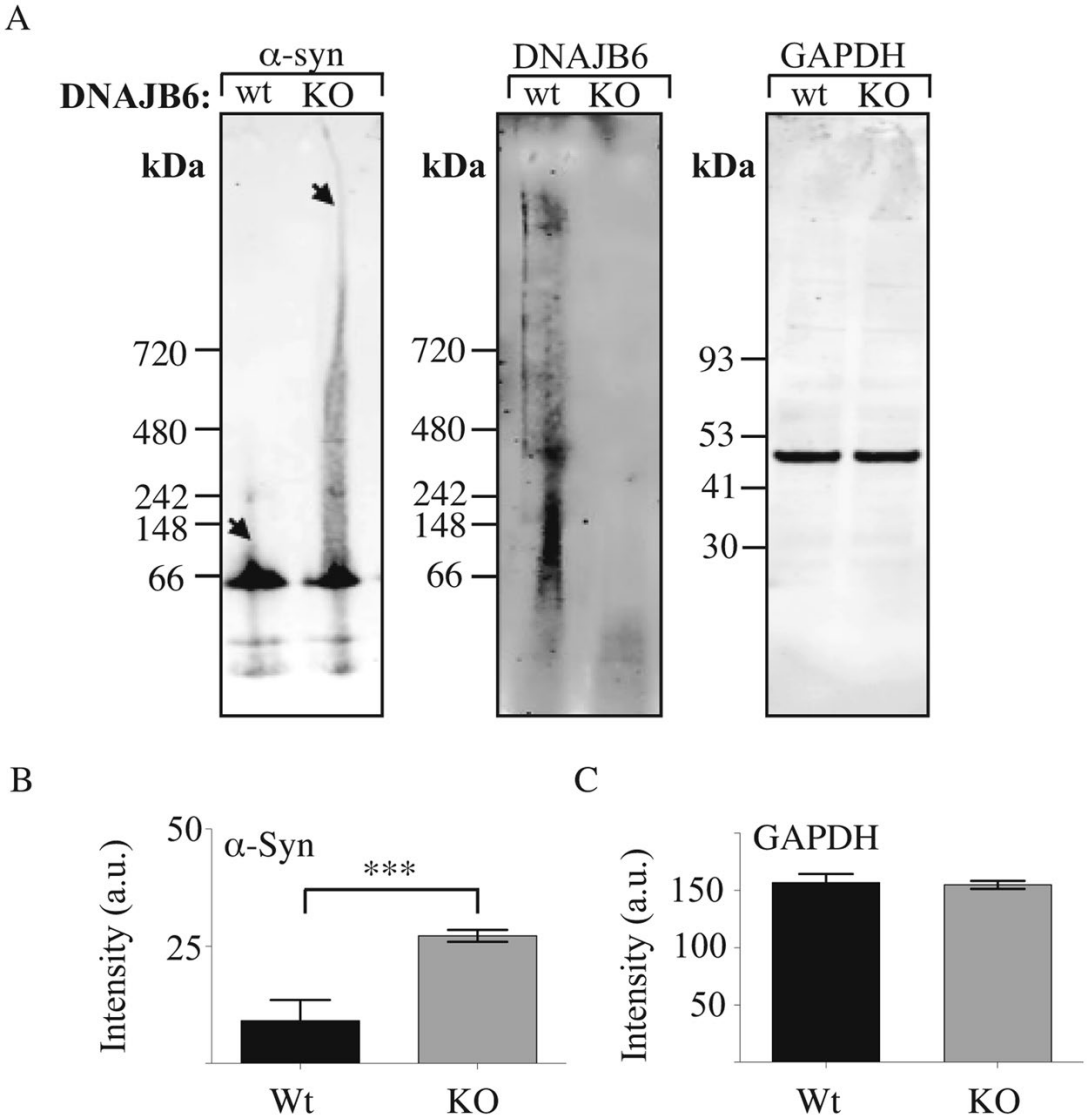
Western blots of native PAGE on soluble proteins in WT and DNAJB6 KO cells. (**A**) From left to right, representative western blots probed against α-synuclein and DNAJB6. As loading control, same samples were also analysed by SDS-PAGE and probed against the protein GADPH. (**B**) Densitometry analysis of the western blots probed against α-synuclein (n = 5) and (**C**) Densitometry analysis of the western blots probed against GADPH (n = 5). Statistical analysis was performed by one-way ANOVA. ***P < 0.001.

To explore if the red puncta observed by fluorescence microscopy would correspond to increased α-syn-DsRed aggregation, lysates from these cells were loaded onto native gels and analyzed by western blotting. These experiments showed that α-syn-DsRed is indeed found to a large extent in a multimeric form in the DNAJB6 KO cells and to a much smaller extent in parental control cells (Fig. 3).

**Figure 4:**
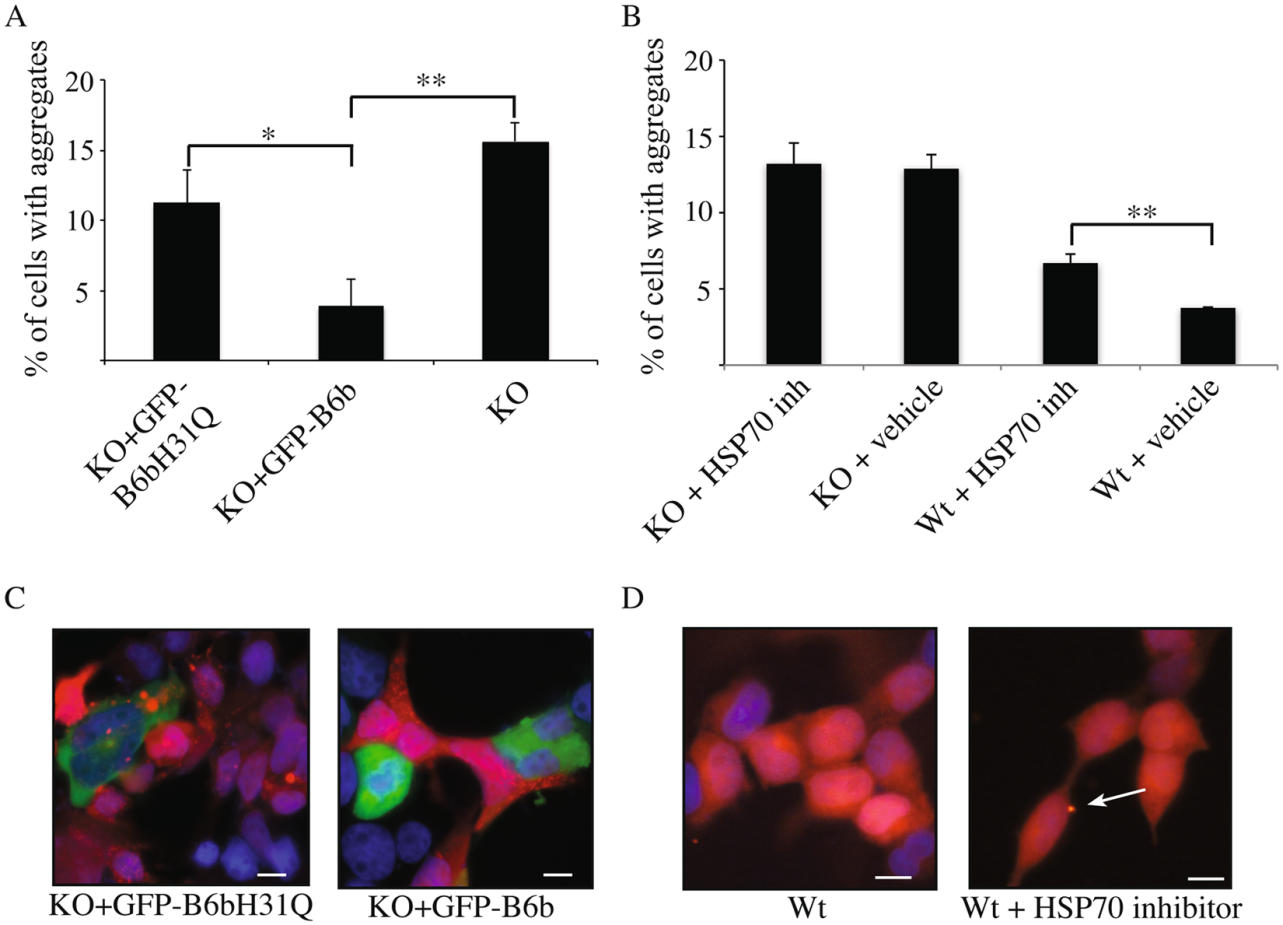
Increased aggregation caused by DNAJB6 KO is dependent on the catalytic function of the DnaJ domain. (**A**) Quantification of aggregation in KO cells transfected with GFP-DNAJB6 wild-type expression plasmid relative to KO cells transfected with GFP-DNAJB6-H31Q expression plasmid (n = 3). (**B**) Quantification of aggregation in WT and KO cells incubated with Hsp70 inhibitor VER-155008 at 10 uM for 2 hours, compared to cells incubated with vehicle control (n = 3). Statistical analysis was performed by One-Way ANOVA. *P < 0.05, **P > 0.01. (**C**) Representative pictures despicting that wt DNAJB6b but not H31Q mutant DNAJB6b suppress α-syn aggregation by flourescnece microscopy. (**D**) Representative pictures demonstrating that increased number of α-syn aggregates were observed when the α-syn-DsRed HEK293T cells were incubated with the HSP70 inhibitor VER-155008 at 10 uM for 2 hours. Scalebar: 10 μM.

J-domain co-chaperones recruit misfolded/unfolded proteins and transfer them to the Hsp70 chaperones by promoting ATP-hydrolysis-dependent transition of the latter to a high affinity state^23^. To investigate if the suppression of α-syn aggregation was dependent on this catalytic activity of the J-domain of DNAJB6, we created a H31Q mutant DNAJB6b, which is unable to transfer unfolded/misfolded proteins to Hsp70^24^. We observed that this mutant could not suppress α-syn aggregation in the KO cells, as the amount of red puncta seen in KO cells expressing mutant DNAJB6 was not significantly different from the non-transfected KO cells (Fig. 4A). To explore this point further, we tested if incubation of WT or DNAJB6 KO cells with a Hsp70 inhibitor would increase the amount of aggregates. Interestingly, an increase in amount of aggregates was only seen in WT cells incubated with the inhibitor, but not in DNAJB6KO cells (Fig. 4B). It has previously been reported that DNAJB6b can inhibit polyQ aggregation and that this effect is dependent on the peptide binding domain DNAJB6b. We therefore wanted to explore if a mutant (mt) DNAJB6b with an impaired peptide binding domain, caused by a series of Ser/Thr to Ala mutations^19^, could inhibit aggregation of α-syn. The mutant DNAJB6-M3 did however cause almost a complete rescue of suppression of α-syn aggregation, when introduced in to DNAJB6KO cells (Supplementary Figure 3a), despite that this mutant protein was not as highly expressed as WT or mt H31Q DNAJB6, when re-introduced in HEK293T cells (Supplementary Figure 3b). Previously this peptide binding domain, mutated in the M3 construct, had been shown to be crucial for inhibiting polyQ aggregation^19^, suggesting that perhaps the mechanism for inhibition of α-syn aggregation is slightly different from that of polyQ. To explore if suppression of α-syn aggregation is specific to DNAJB6, we reintroduced another DNAJB protein, DNAJB8, into DNAJB6 KO cells and analyzed the effect on α-syn aggregation. Introduction of DNAJB8 did cause a partial suppression of α-syn aggregation. This result shows that while DNAJB8 can suppress α-syn aggregation to a smaller extent upon overexpression, DNAJB6 is a more potent suppressor of α-syn aggregation (Supplementary Figure 3c and d). Interestingly, polyQ aggregation in WT relative to DNAJB6 KO cells did not differ significantly, suggesting that perhaps DNAJB6 does not suppress aggregation of all amyloid proteins (Supplementary Figure 4).

**Figure 5:**
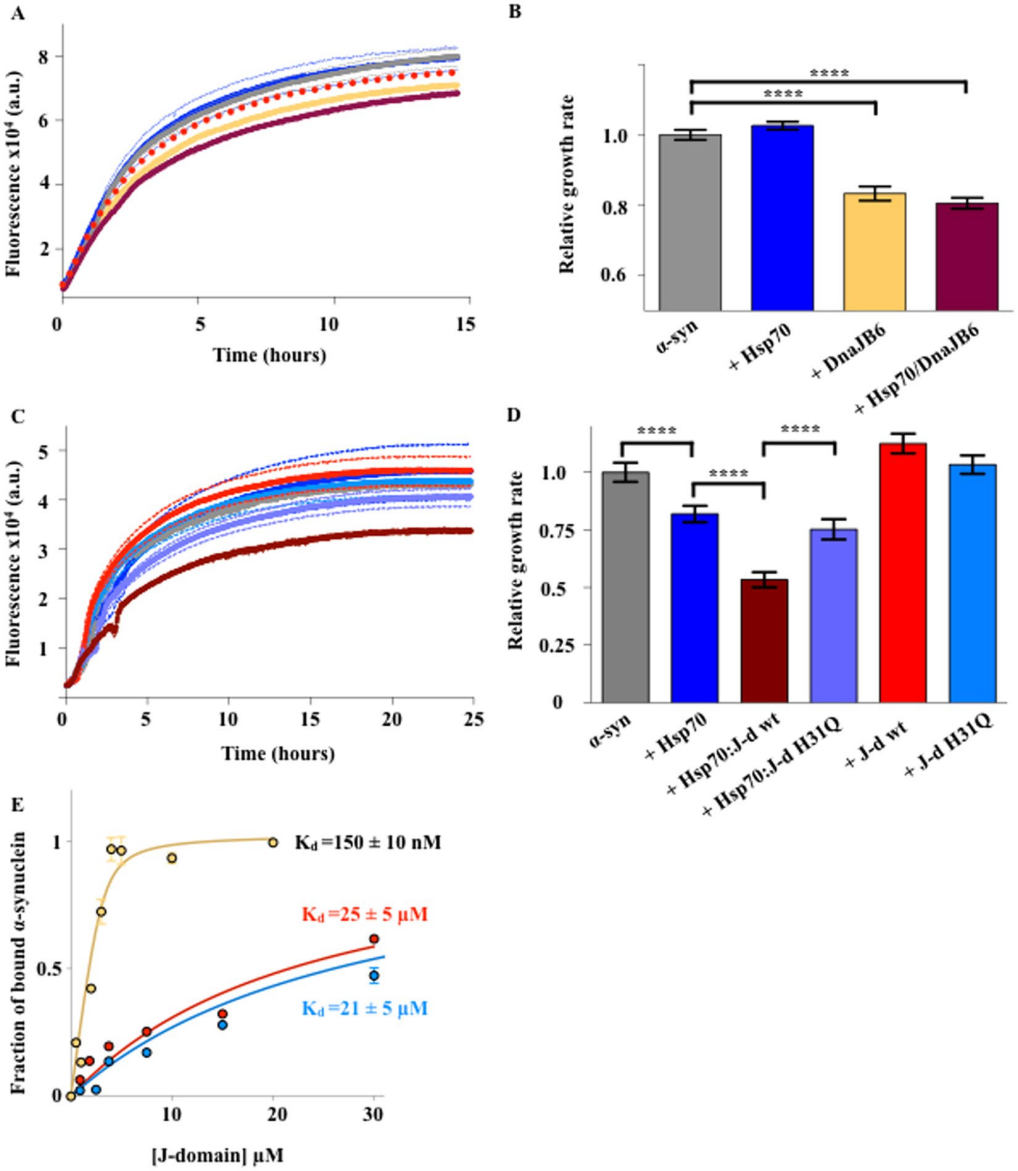
Effect of DNAJB6 on the aggregation of α-syn. (**A**) Seeded aggregations of α-syn alone (grey) or in the presence of either 0.5 µM Hsp70 (blue), 0.15 µM DNAJB6 (yellow), or 0.4 µM Hsp70 and 0.1µM DNAJB6 (maroon). Individual values are the mean of four independent experiments. The red dashed line is the sum of the concentration-corrected effects derived from the blue and yellow lines, which corresponds to the theoretical aggregation profile in the presence of 0.4 µM Hsp70 and 0.1 µM DnaJB6 if their effect was simply additive. (**B**) Bar plot of the initial velocities of the aggregation reactions in (**A**) normalised on the value of α-syn alone. (**C**) ThT aggregation experiments in seeding conditions of α-syn in the absence (grey) or in the presence of either 0.5 µM Hsp70 (blue), wt J-domain (red), H31Q J-domain (cyan), Hsp70:wt J-domain (maroon) or Hsp70:H31Q J-domain (violet). (**D**) Bar plot representing the initial growth rates of aggregation from panel (C) normalised on the value of α-syn alone. (**E**) Fluorescence binding assay of DnaJB6 (yellow), wt J-domain (red), or H31Q J-domain (cyan), to Alexa488-α-synuclein. The apparent K_d_s of binding are reported on the side of each curve. Statistical analysis was performed by one-way ANOVA with post-multiple comparison (99% CI, *P ≤ 0.05, **P ≤ 0.01, ***P ≤ 0.001, ****P ≤ 0.0001).

To gauge the role of Hsp70 in the DNAJB6-mediated suppression of α-syn aggregation, we turned to an *in vitro* thioflavin T (ThT)-based aggregation assay. In particular, we tested the aggregation of α-syn in the absence (control condition) and in the presence of 0.5 μM Hsp70, or 0.13 μM full length DNAJB6 (for simplicity we will refer to this protein variant as simply “DNAJB6”) or a combination of 0.4 μM Hsp70 and 0.1 μM DNAJB6. (Fig. 5a, b). These experiments revealed that the addition of Hsp70 in combination with DNAJB6 more strongly inhibits the initial growth rate of aggregation than the presence of Hsp70 alone, given that the total concentration of chaperone molecules was equal in the two conditions. Furthermore, the aggregation profile obtained in the presence of both Hsp70 and DNAJB6 was significantly different from the one expected assuming a simply additive effect (Figure 5a), suggesting a cooperative interaction between the two proteins.

In order to further validate our findings and to determine whether DNAJB6 stimulates the anti-aggregation activity of Hsp70 by also promoting its ATPase activity, we tested the anti-aggregation activity of  isolated J-domain variants (Figure 5c, d)These proteins have a weaker binding to α-syn with respect to DNAJB6 (Figure 5e) and Hsp70^25^ and they can be used for selectively studying the effect of the stimulation of the ATPase activity. In particular, we performed seeded ThT aggregation experiments of α-syn alone, α-syn with 0.5 μM Hsp70, α-syn with Hsp70 and either the wild-type or the H31Q DNAJB6 J-domain, the last acting as a dominant negative by failing to activate Hsp70 ATPase activity. We noticed that the addition of Hsp70 in combination with the wild-type DNAJB6 J-domain was able to produce a decrease of the initial growth rate of aggregation by 50% (Figure 5c, d), which was the strongest inhibition observed in these assays. In contrast, the H31Q J-domain in presence of Hsp70 was much less effective, producing a reduction of the initial growth rate of aggregation by only 25% (Figure 5d), which was close to the effect caused by Hsp70 alone, that resulted in a reduction of the initial growth rate of aggregation by 18%.

As a validation of the ThT aggregation assays, we performed dot blot experiments to quantify the final amount of monomer in solution in the absence or presence of different combinations of Hsp70 and DNAJB6 after 15 h of aggregation (Supplementary Figure 5). Also in this case, the combination of the two chaperone molecules was able to produce a stronger inhibition of α-syn aggregation than all the other conditions. Furthermore, the final amount of monomer in solution very well correlated with the fluorescence of the ThT at the same time points of aggregation, validating the quantitative analysis extrapolated from the ThT fluorescence experiments (Supplementary Figure 5c).  These results taken together demonstrate that DNAJB6 facilitates the inhibition of α-syn aggregation mediated by Hsp70 in a synergistic manner, since the effect was stronger for DNAJB6 and Hsp70 together than of Hsp70 alone or together with a mutant DNAJB6, which was unable to catalitically stimulate Hsp70.

## Discussion

In this work we have shown that the co-chaperone DNAJB6 in combination with its partner Hsp70 is particularly effective in preventing the aggregation of α-syn. In particular, our results indicate that KO of the gene encoding for DNAJB6 leads to the formation of an increased amount of α-syn aggregates in a cell line overexpressing α-syn (Fig. 2). In addition, we have shown that inhibition of α-syn aggregation mediated by DNAJB6 depends specifically on the activity of the J-domain, which catalyzes the transfer of misfolded or unfolded proteins to Hsp70^24^ (Fig. 4). It is most likely only DNAJB6b that suppress α-syn aggregation, as elimination of DNAJB6a did not cause an increase in α-syn aggregation (Supplementary Figure 2). The peptide binding domain impaired mutant (DNAJB6-M3) partially rescued α-syn aggregation (Supplementary Figure 3), when transfected back into KO cells. This was surprising, but could be explained by DNAJB6-M3 retaining a weak affinity for α-syn, sufficient to suppress aggregation of the protein.

The finding of DNAJB6/Hsp70 mediated suppression of α-syn aggregation in cells, was consolidated by an *in vitro* approach, using a ThT aggregation assay to measure the aggregation rate of α-syn in presence of recombinant Hsp70 and wild-type and H31Q mutant DNAJB6 forms (Fig. 5). In this experimental set up, we employed a low concentration of recombinant Hsp70 (0.5 μM, corresponding to a molar ratio Hsp70 to monomeric α-syn of 1:140) in order to minimize the effects of Hsp70 on the aggregation of α-syn. The addition of wild-type DNAJB6 to the mixture is able to produce a significant decrease in the initial velocity of the aggregation reaction, which proves that the anti-aggregation activity of Hsp70 is strongly stimulated by DNAJB6. By contrast, under the same experimental conditions, the mutant H31Q does not show any significant effect. Taken together, these results indicate that DNAJB6 has a key role in maintaining α-syn in its soluble state and in preventing its aggregation in cells, as its KO alone is able to produce an increase of 15% in the number of cells with aggregates with respect to the control conditions. To do so, DNAJB6 cooperates with Hsp70 by functioning as a molecular chaperone, which binds and presents cargo α-syn to Hsp70.

Hsp70 is an important component of the protein homeostasis system, which has been shown to be able to inhibit the aggregation of α-syn *in vitro* and *in vivo* ^12, 14^. These results, however, identified also a missing link in the picture, as it is known that Hsp70 recruits unfolded or misfolded proteins via co-chaperones, which have not been identified yet in the case of α-syn^24^. As DNAJB6 is highly expressed in neurons and brain tissues^26^, it may play a role in determining the interaction network of Hsp70 in the different cell types and compartments of the central nervous system^16^. Indeed, immunohistochemistry and real time PCR analysis on post mortem brain tissues of PD patients showed that DNAJB6 is present in LBs^16^. This finding suggests that dysregulation of DNAJB6 may contribute to the onset of PD and implies that DNAJB6 is able to interact with α-syn *in vivo*, although this hypothesis remains to be explored.

DNAJB6 has been shown to suppress aggregation of other amyloid proteins as well such as Aβ and polyQ (huntingtin)^17–20^, suggesting that DNAJB6 could have a more general protective role against many different types of protein misfolding and aggregation diseases. However, these other studies where purely based on the overexpression of DNAJB6, and in our studies we did not see increased aggregation of polyQ-GFP in cells that did not express DNAJB6, which indicates that endogenous levels of DNAJB6 do not suppress polyQ aggregation in HEK293T cells (Supplementary Figure 4). It may also be worth looking into whether other DnaJ proteins might affect α-syn aggregation as well. For example, it is intriguing that mutations in two DnaJ encoding genes have been linked to PD^27, 28^.

## Conclusions

We have identified the combination of DNAJB6 and Hsp70 as an effective molecular chaperone system for the prevention of α-syn aggregation in cells. As this system is known to have many other substrates, our findings suggest that it may affect aggregation of other aggregation-prone proteins. Future studies will show whether these results could give rise to novel avenues for the development of drugs that mimic or increase the efficiency of the protein homeostasis system through the modulation of the combined activity of DNAJB6 and Hsp70.

## Materials and Methods

### Cell lines, western blotting, antibodies and reagents

HEK293T cells were grown in DMEM (Sigma-Aldrich) plus 10% FBS (Sigma-Aldrich) and penicillin/streptomycin (P/S). Proteins were first separated on 10 or 12% polyacrylamide gels and then subjected to Westernblot transfer. The membranes were blocked with 5% (w/v) dried skimmed milk in TBS (25 mM Tris-HCl pH 7.5, 150 mM NaCl). Primary antibodies used for Western blotting or ICC were anti-α-syn (BD, Cat# 610787), HRP coupled anti-Actin (Sigma, Cat# A3854), anti-HSP70 (AbCam, Cat#: ab47455), anti-GFP (Thermo Fischer, Cat#: MA5-15256), anti-Lamin-B1(AbCam, Cat#: ab16048), anti-pan cytokeratin (Thermo Fischer, Cat# MA5-13203) and anti-DNAJB6 (AbCam, cat# ab198995). Secondary antibodies used were anti-mouse HRP-conjugated antibodies (Dako), and RDye fluorescently labeled secondary antibodies (Li-Cor, UK). VER-155008 (Hsp70 inhibitor) was purchased from Sigma-Aldrich.

### Plasmids, Fluorescence microscopy and Immonocytochemistry

DNAJB6 was inserted into pAcGFP1-C1 vector (Clontech) by amplifying human DNAJB6 cDNA with primers containing XhoI and SacII restriction site overhangs respectively: 5′ctgactcgagctatggtggattactatgaagttctaggc 3′ and 5′ ctagccgcggttacttgttat ccaagcgcagcagctgctccttaccatttattgttaaggactttaactggccatcttc 3′. For insertion of J-domain only (codon 1-69) into pAcGFP1-C1 vector, the following primers were used containing XhoI and sacII restriction site overhangs: 5′ctgactcgagctatggtggattactatgaagttctaggc 3′ and 5′ actgccgcggttagccatatttgtcataga tgtcccgtttcttagc 3′. For generation DNAJB6 H31Q mutant construct the following primers were used: 5′ ctggcactgaagtggcatccagataaaaatcctgagaataaag 3′ and 5′ ctcaggatttttatctggatgccacttcagtgccagtttccga 3′. Plasmid containing DNAJB6-M3 cDNA, was a kind gift from Professor Harm Kampinga (University of Groningen). The cDNA was amplified with same primers as full length DNAJB6 for insertion into pAcGFP1-C1. PolyQ (74Q) into pEGFP for mammalian expression of PolyQ-GFP fusion protein, was acquired from Addgene (plasmid# 40262). For transfection of the respective constructs into HEK293T-α-syn-dsred cells, cells were transfected using Lipofectamine Ltx method (Thermo Fischer) for 16 hours, then medium was changed to normal medium, followed by 24 hours incubation period before fixing the cells.

For quantification of α-syn-dsred aggregates in HEK293T-α-syn-dsred cells, cells were washed with PBS fixed for 15 minutes in 4% Paraformaldehyde (PFA), washed with PBS, subsequently incubated with PBS + höechst for 3 minutes for nuclear staining, where after the cells where washed with PBS again. For each independent experiment at least 200 cells were counted and analyzed for the presence or absence of red puncta. We noted that the sequence of DNAJB6b used in experiments of  Figures 1, 2 and 4 contained the mutation F46S, which does not affect DNAJB6b localisation and ability to inhibit α-syn aggregation in HEK293T-α-syn-dsred cells. For staining with anti-DNAJB6 cells fixed for 15 minutes in PFA, washed ×2 in PBS, permeabilized with Tris buffered Saline (PBS) + 0,5% Triton X-100 for 5 minutes, followed by washing to times with PBS, subsequently blocked for 1 hour with PBS + 5% Bovine Serum Albumine (BSA), then incubated in PBS + 5% BSA + 1:1000 rabbit anti-DNAJB6 antibody for 1 hour, washed 3 times with PBS incubated with 1:500 anti-rabbit coupled dylight 649 antibody in TBS + 5% BSA, washed ×3 and stored at +4° until analysis by fluorescence microscopy.

### CRISPR/Cas9 guide design

For design of guide sequences for CRISPR/Cas9 targeted indels in the DNAJB6 gene the sanger programme was used: http://www.sanger.ac.uk/htgt/wge/find_crisprs. The sequences used for the guides were: 5′ CACCgGGAGGCATATGAAGTGCTGT 3′ and 5′ aaacAGATATCGGAAACTGGCACTc 3′, targeting the beginning of exon 3. Cloning intoGFP-CAS9 construct (Addgene: cat# 48138) and transformation of HEK293T- α-syn-dsred cells were done according to protocols described in ref. 21. GFP-CAS9 positive cells were single cell sorted into 96 well plates after 36 hours and single cell clones were analyzed for DNAJB6 KO by western blot analysis. For design of guide sequences for CRISPR/Cas9 targeted indels in the DNAJB6 gene in the start of the first DNAJBa specific exon (exon 8), the sequences used for guides were 5′ CACCGTCCTACAGAATTGTCGAGAA 3′ and 5′ AAACTTCTCGAC AATTCTGTAGGAC 3′.

### Production of recombinant protein

N-terminally GST-tagged human full length DNAJB6 and WT or H31Q mutant DNAJB6 J-domain (amino acid residue 1–69 of human DNAJB6) cDNA were inserted into a pGEX-4T1 vector by using the following primers containing BamHI and XhoI overhangs respectively: Full length DNAJB6 cDNA: 5′ actgggatccatggtg gattactatgaagttctaggcgtg 3′ and 5′ actgctcgagttacttgttatccaagcgcagcagctg 3′. DNAJB6 J-domain: 5′ actgggatccatggtggattactatgaagttctaggcgtg 3′ and 5′ actgctcgagttagccata tttgtcatagatgtcccgtttcttagc 3′. Plasmids were transformed into BL21 C3013, cells (New England BioLabs UK). Bacterial cultures were grown at 37 °C in LB medium containing100 mg/ml ampicillin to OD600 nm 0.8, shifted to 20 °C and expression was induced with 0.1 mM IPTG for 16 hr. Afterwards, the cells were harvested and lysed with a high-pressure homogenizer (EmulsiFlex-C3; Avestin) in buffer A [50 mM Tris-HCl pH 7.5, 500 mM NaCl, 1 mM MgCl_2_, 0.2% (v/v) Triton X-100, 10% (v/v) glycerol, 20 mM imidazole] containing protease inhibitors (2 mM PMSF, 4 mg/ml pepstatin, 4 mg/ml leupeptin, 8 mg/ml aprotinin) and 0.1 mg/ml DNaseI. The lysates were cleared by centrifugation (30 min at 25,000 g) and incubated with 0.7 ml GSH-Sepharose beads per 1 l of expression culture for 2 hr at 4 °C. The beads were transferred to a column and washed with 20 ml wash buffer B [50 mM Tris-HCl pH 7.5, 500 mM NaCl, 1 mM DTT, 0.2% (v/v) Triton X-100, 10% (v/v) glycerol] containing protease inhibitors, 20 ml wash buffer C [50 mM Tris-HCl pH 7.5, 300 mM NaCl, 10 mM MgCl_2_, 1 mM DTT, 0.1% (v/v) Triton X-100, 10% (v/v) glycerol] containing protease inhibitors and 20 ml wash buffer C sequentially supplemented with (i) 1% (v/v) Triton X-100, (ii) 1 M NaCl, (iii) 3 mMATP, (iv) or 0.5 M Tris-HCl pH 7.5. Bound proteins were then eluted in elution buffer [50 mM HEPES-KOH pH 7.4, 100 mM KCl, 4 mM MgCl_2_, 1 mM CaCl_2_, 0.1% (v/v) Triton X-100, 10% (v/v) glycerol, 40 mM reduced glutathione]. For buffer exchange the proteins were loaded onto Centri Pure P10, Zetadex Gel Filtration columns (Emp Biotech) which had been equilibrated with HKM buffer (50 mM HEPES-KOH pH 7.4, 150 mM KCl, 10 mM MgCl_2_) + 1 mM DTT buffer and proteins were eluted in HKM buffer + 1 mM DTT as well.

Human α-syn wild type and A90C mutant variants were expressed and purified as a monomeric fraction from *E. coli* BL21 (DE3) Gold Strain (Agilent Technologies, Santa Clara, USA) as described previously^29^. Recombinant N-hexa-His-tagged Hsp70 (human Hsp70 1 A, GenBank ID: NP005336) was overexpressed from pET-28b vector (Merck KGaA, Darmstadt, Germany) in *E. coli* BL21 (DE3) Gold Strain (Agilent Technologies, Santa Clara, USA) and purified by affinity chromatography and size exclusion as previously described^30^. Protein purity, as determined by SDS–PAGE, exceeded 95%.

Solutions of the purified proteins were then divided into aliquots, flash-frozen in liquid nitrogen and stored at −80 °C; each protein aliquot was thawed only once before use. Protein concentrations were determined by absorbance measurements at 280 nm using theoretical extinction coefficients calculated with Expasy ProtParam.

### Preparation of α-syn seeds

300 µl Solutions containing 70 μM monomeric α-syn in 50 mM Tris pH 7.4, 150 mM KCl, 5 mM MgCl_2_, 0.01% NaN_3_ were incubated at 37 °C under constant shaking at 200 rpm for 4 days. The fibrillar pellets were centrifuged (16000 × g, 30 min) and washed twice with 300 µl of buffer. Fibrils were then resuspended at a concentration of 100 μM and sonicated (1 min, 10% max power, 30% cycles) using a probe sonicator (Bandelin, Sonopuls HD 2070, Bandelin Elec., Germany) in order to produce first generation fibrils. Second generation fibrils were prepared by incubating 100 μM monomeric α-syn in the presence of 10 μM first generation fibrils in 50 mM Tris pH 7.4, 150 mM KCl, 5 mM MgCl_2_, 0.01% NaN_3_ (500 µl) at 37 °C under quiescent conditions for 13–14 h. The suspension was finally sonicated (20 s, 10% max power, 30% cycles).

### Seeded aggregation assays

Solutions of 70 μM monomer and 5% seeds of α-syn alone or in the presence of different concentrations of Hsp70, wild-type or H31Q DNAJB6 J-domain and DNAJB6 full length in 50 mM Tris pH 7.4, 150 mM KCl, 5 mM MgCl_2_, 0.01% NaN_3_, 5 mM ATP, 20 μM ThT were incubated at 37 °C in quiescence. ThT fluorescence was monitored in low-binding, clear-bottomed half-area 96-well plates (Corning Inc., New York, NY, USA). Emissions at 480 nm was recorded every 2 min with excitation at 440 nm, using a CLARIOstar plate reader (BMG Labtech, Ortenberg, Germany).

### Dot blot experiments

Solutions of 70 μM monomer and 5% seeds of α-syn alone or in the presence of 0.5 μM Hsp70, or 0.5 μM DNAJB6, or 0.15 μM DNAJB6, or 0.35 μM Hsp70 and 0.15 μM DNAJB6 in 50 mM Tris pH 7.4, 150 mM KCl, 5 mM MgCl_2_, 0.01% NaN_3_, 5 mM ATP were incubated for 15 h at 37 °C in quiescence. Samples were then centrifuged at 120,000 rpm at 10 °C for 1 h using a TLA-120.2 Beckman rotor. Samples of 3 μl of the supernatants were then spotted on a 0.45 μm pore size nitrocellulose membrane (Amersham Bioscience, Little Chalfont, UK) and let dry for 1 h RT.

The membrane was blocked in PBS + 5% (w/v) BSA for 1 h RT and then incubated in PBS + 5% (w/v) BSA + 1:2000 mouse anti α-syn polyclonal antibody (Transduction Laboratories, Lexington, KY, USA) ON at 4 °C. After incubation with primary antibody, it was washed in PBS + 0.02% Tween-20 three times, 15 min each time, and incubated in PBS + 5% (w/v) BSA + 0.02% Tween-20 + 1:2000 goat anti-mouse Alexa Fluor-488 secondary antibody (Thermo Fisher Scientific, Waltham, MA, USA) for 1 h at RT. Finally, the membrane was washed 3 times in PBS + 0.02% Tween-20 and the fluorescence reviled by utilizing a Typhoon Trio scanner (Amersham Bioscience, Little Chalfont, UK).

### Native electrophoresis

Native electrophoresis analysis was performed on 10 μg samples, using NativePAGE™ Bis-Tris 4–16% precast minigel system (Life Technologies, Carlsbad, CA, USA), according to manufacturer instruction. Proteins were blotted on a PVDF membrane by using an iBlot Dry Blotting System (Life Technologies, Carlsbad, CA, USA). Membranes were incubated in PBS + 5% (w/v) BSA for 1 h at RT and then probed overnight at 4 °C with either anti- α-syn (Transduction Laboratories, Lexington, Kentucky, USA) or anti- DnaJB6 (Abcam, Cambridge, UK) at 1∶2000 dilution in PBS + 5% (w/v) BSA. After incubation in primary antibody, membranes were washed thrice in PBS + 0.02% Tween-20 for 15 min each time, and subsequently incubated for 1 h at room temperature in goat anti-mouse Alexa488-conjugated secondary antibody (Life Technologies, Carlsbad, CA, USA) at 1∶2000 dilution. Control western blotting to verify that equal protein amounts were used for the analysis was performed on a NuPAGE loaded with the same samples used for the NativePAGE analysis. The membrane was then probed with a rabbit polyclonal anti-GAPDH antibody (Abcam, Cambridge, UK) and a goat anti-rabbit Alexa488-conjugated secondary antibody (Life Technologies, Carlsbad, CA, USA) both at a 1:2000 dilution in blocking solution using the protocol described above.

### Labeling reaction of α-synuclein

A90C α-syn was labeled with Alexa Fluor 488 C5 maleimide (Thermo Fisher Scientific, Waltham, MA, USA) via the cysteine thiol moiety. The protein was incubated in the presence of a 5 molar equivalent excess of the dye in PBS for 3 h at room temperature in the dark. The labeled protein was then purified from the excess of free dye by a P10 desalting column containing a Sephadex G25 matrix (GE Healthcare, Little Chalfont, UK), divided into aliquots, flash frozen and stored at −80 °C. Each aliquot was thawed immediately prior to use. The labeling efficiency was more than 95% as estimated by mass spectrometry. The labeled protein concentration was estimated by absorbance measurement at 495 nm using the extinction coefficient of the free dye (73,000 M^−1^ cm^−1^).

### Fluorescence titration with monomeric α-synuclein

Fluorescence titration experiments were carried out by incubating 2 μM Alexa488- α-syn in 50 mM Tris pH 7.4, 150 mM KCl, 5 mM MgCl_2_ for 30 min at 25 °C in the presence of different concentrations of the chaperone. Fluorescence emission spectra were recorded using a CLARIOstar plate reader (BMG Labtech, Ortenberg, Germany). The increase in fluorescence intensity at the emission maximum was plotted as a function of Hsp70 concentration, and analysed assuming a one site binding model using the following equation:

$$F=\frac{{F}_{max}-[DnaJB6]}{{K}_{d}+[DnaJB6]}$$

where F is the fluorescence intensity observed at a given concentration of free DnaJB6 in equilibrium (for practical reasons this was approximated to be the total Hsp70 concentration), F_max_ is the fluorescence intensity at saturation and K_d_ is the apparent dissociation constant of the complex. The fraction of bound α-syn was plotted as a function of protein concentration in order to compare affinities between the different chaperone variants.

## Electronic supplementary material

Supplementary figures
