# Author Correction: The molecular chaperones DNAJB6 and Hsp70 cooperate to suppress α-synuclein aggregation

**DOI:** 10.1038/s41598-018-29893-7

**Published:** 2018-08-02

**Authors:** Francesco A. Aprile, Emma Källstig, Galina Limorenko, Michele Vendruscolo, David Ron, Christian Hansen

**Affiliations:** 1 Department of Chemistry, University of Cambridge, Cambridge, CB2 1EW UK; 2 Cambridge Institute for Medical Research, University of Cambridge, Cambridge, CB2 0XY UK; 3 Molecular Neurobiology, Department of Experimental Medical Science, BMC B11, 221 84, Lund, Sweden

Correction to: *Scientific Reports* 10.1038/s41598-017-08324-z, published online 22 August 2017

The Article mentions but does not include control data demonstrating that a random mutation in the cDNA sequence of DNAJB6b used in the study does not affect the behaviour of its protein product. Specifically, the cDNA sequence of DNAJB6b WT and DNAJB6b H31Q in the plasmids used for all the experiments reported in this study contained a mutation (TTC to TCC) leading to the F46S mutation in the translated proteins, which was randomly introduced during the cloning. The mutation did not change the localisation or ability to inhibit α-syn aggregation in HEK293T-α-syn-dsred cells, as shown in Figure 1 below.

**Figure 1:**
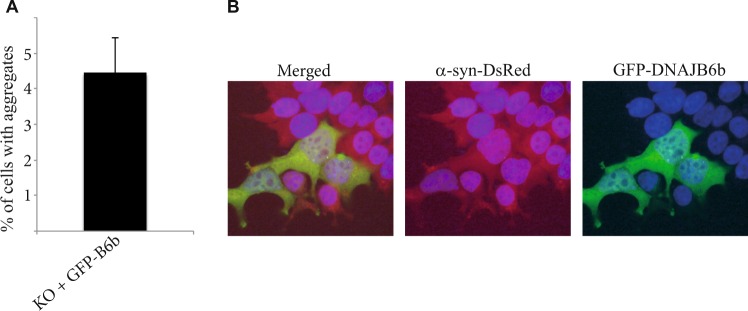
Quantification of aggregates in α-syn-DsRed expressing HEK293 DNAJB6 KO cells transfected with GFP-DNAJB6b expression construct (n = 3) (**A**). Experiment showing that α-syn-DsRed aggregation is suppressed in DNAJB6 KO cells transfected with GFP-DNAJB6b as well as the localization of the GFP-DNAJB6b fusion protein (**B**).

This does not affect the conclusions of the Article.

