# Supplementary material for: The molecular chaperones DNAJB6 and Hsp70 cooperate to suppress α-synuclein aggregation
Source: Sci Rep. 2017 Aug 22;7:9039. doi: 10.1038/s41598-017-08324-z (PMC5567236; doi:10.1038/s41598-017-08324-z)
Supplement: Supplementary file 1 — Supplementary figures [file 41598_2017_8324_MOESM1_ESM.pdf]

Supplementary figures:

The molecular chaperones DNAJB6 and Hsp70 cooperate to suppress α-synuclein aggregation

Francesco A. Aprile[1], Emma Källstig[3], Galina Limorenko[3], Michele Vendruscolo[1], David Ron[2] and Christian Hansen[2,3,*]

[1]Department of Chemistry, University of Cambridge, Cambridge CB2 1EW, UK
[2]Cambridge Institute for Medical Research, University of Cambridge, Cambridge CB2 0XY, UK
[3]Molecular Neurobiology, Department of Experimental Medical Science, BMC B11, 221 84 Lund, Sweden

Supplemental figure 1

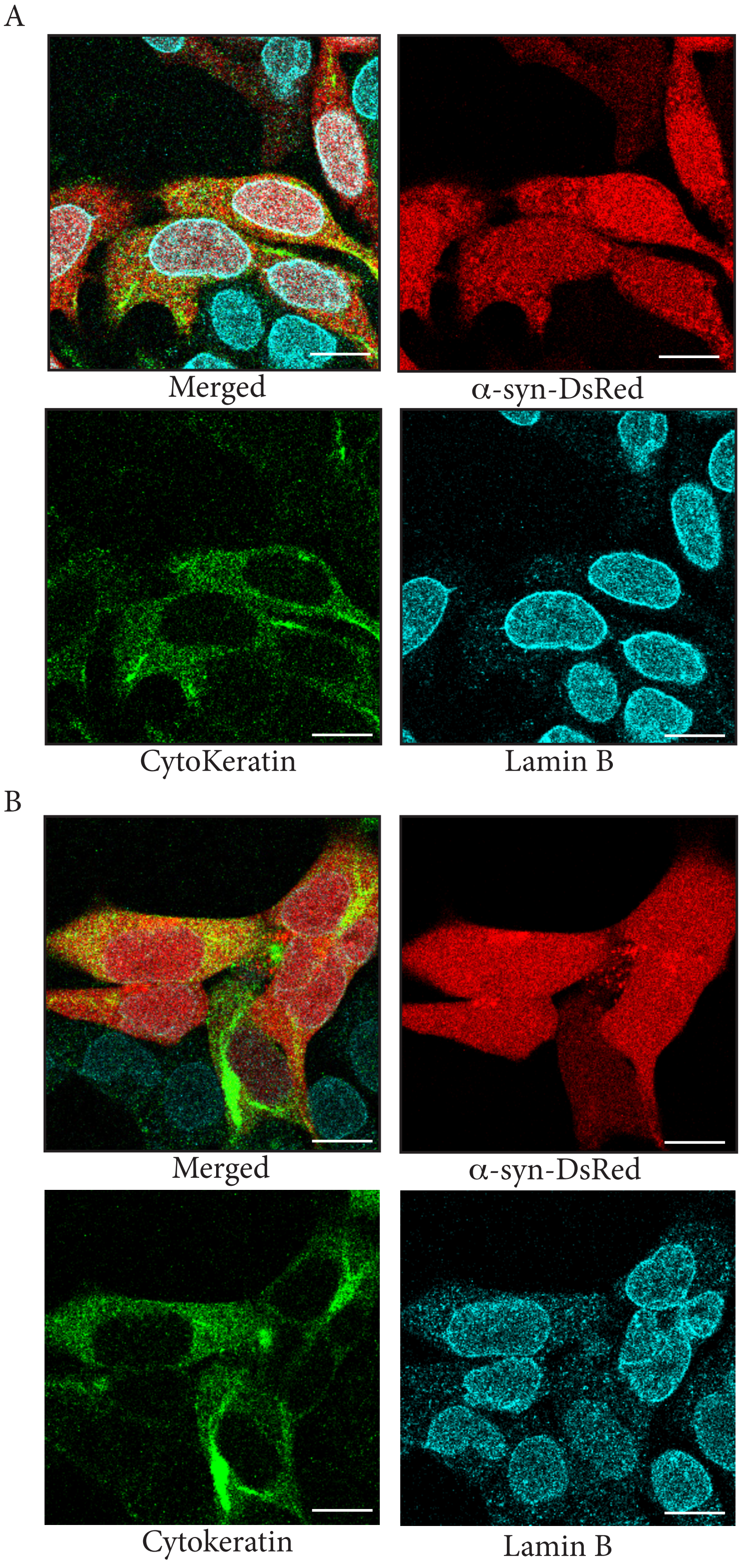

Supplementary Figure 1:α-syn-DsRed is present in both nucleus and cytoplasm of α-syn-DsRed HEK293T cells. α-syn-DsRed HEK293T cells were stained with the nuclear envelope marker Lamin B1 and the cytoskeletal marker Cytokeratin. Nuclear and cytoplasmic localisation of α-synuclein-DsRed is consistent in both (A) Wt and (B) DNAJB6 KO cells. Scalebar: 10 μM

Supplemental figure 2

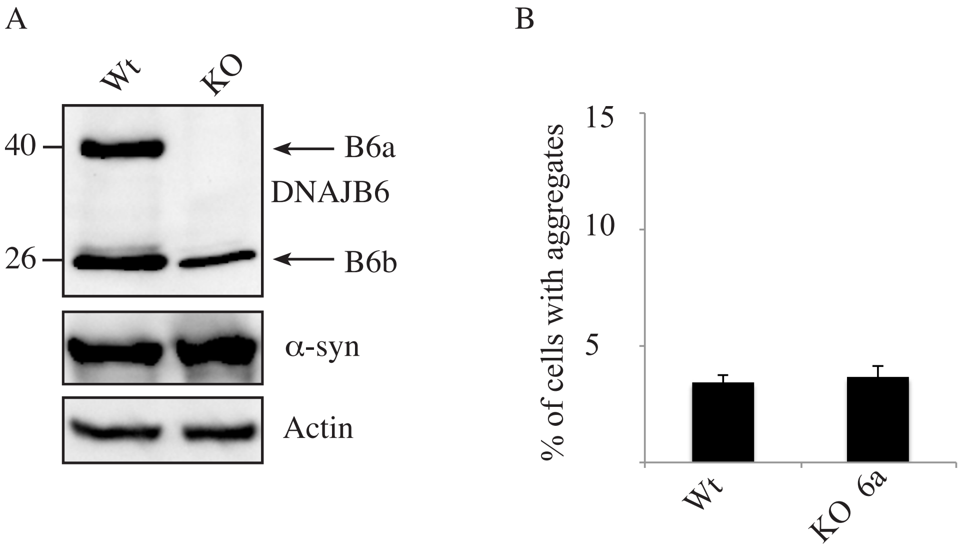

Supplementary figure 2: KO of DNAJB6a isoform does not change aggregation of α-syn. A, Western blot confirming the lack of DNAJB6a in α-syn-DsRed HEK293T cells. Actin was used as a loading control. B, Quantification of α-synuclein aggregates in both Wt and DNAJB6a KO α-synuclein-DsRed HEK293T cells. No significant difference between cell types.

Supplemental figure 3

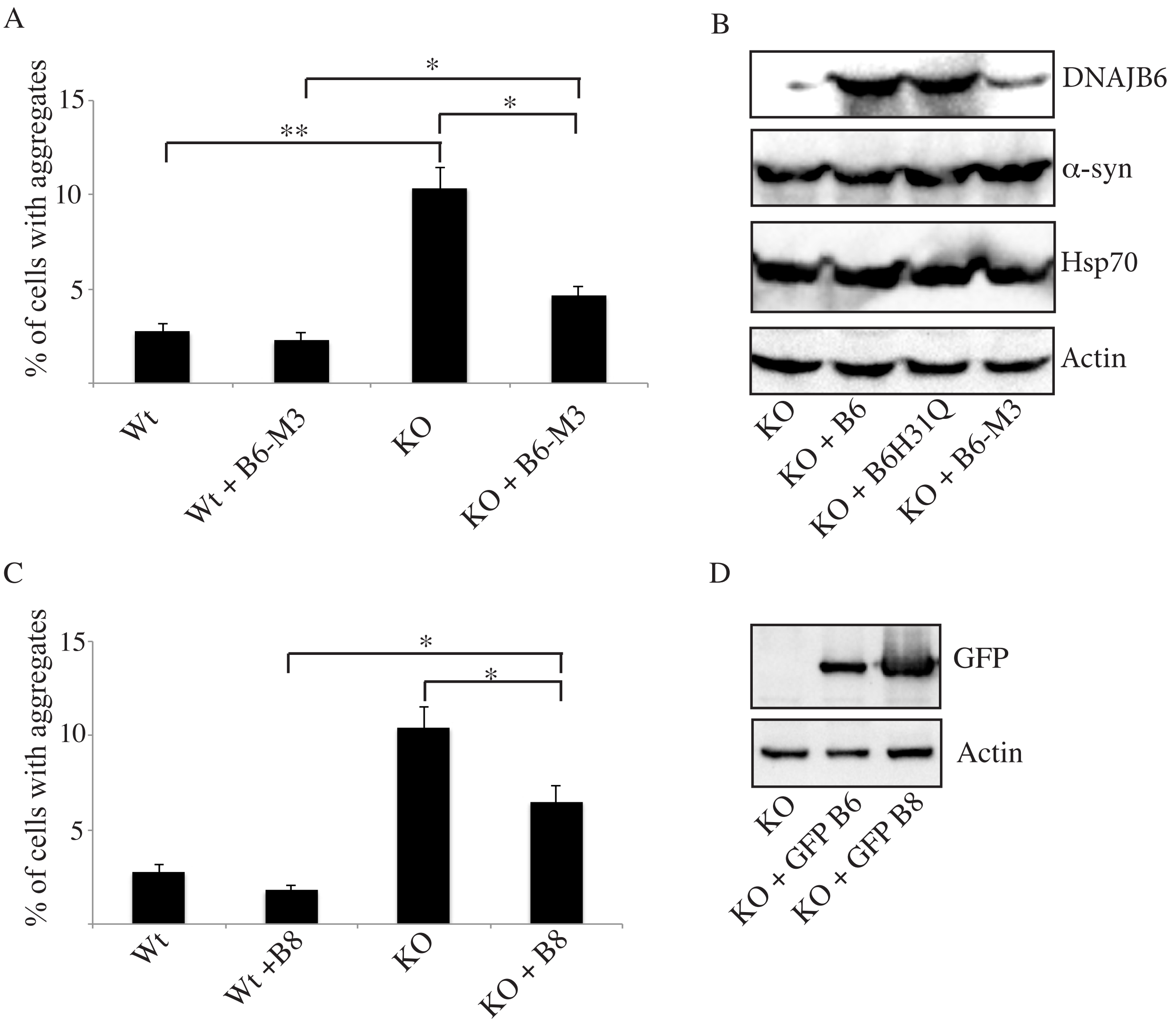

Supplementary Figure 3: DNAJB6-M3 is a potent suppressor of α-synuclein aggregation, whereas DNAJB8 only partially inhibits α-synuclein aggregation. A, Quantification of α-synuclein aggregation in Wt and DNAJB6 KO α-syn-DsRed HEK293T cells, transfected with GFP-DNAJB6-M3 expression plasmid relative non-transfected cells (n=3). B, Western blot analysis of Hsp70, α-synuclein, α-syn and DNAJB6 expression in transfected or non-transfected DNAJB6 KO α-syn-DsRed HEK293T cells. C, Quantification of α-syn aggregation in Wt and KO cells transfected with GFP-DNAJB8 expression plasmid (n=3). D, Western blot analysis of expression of GFP-DNAJB6 relative to GFP-DNAJB8 transfected or non-transfected DNAJB6 KO HEK293T cells analyzed by anti-GFP staining. Statistical analyses were performed by One-way ANOVA. *: $P<0.05$, **: $P<0.01$.

Supplemental figure 4

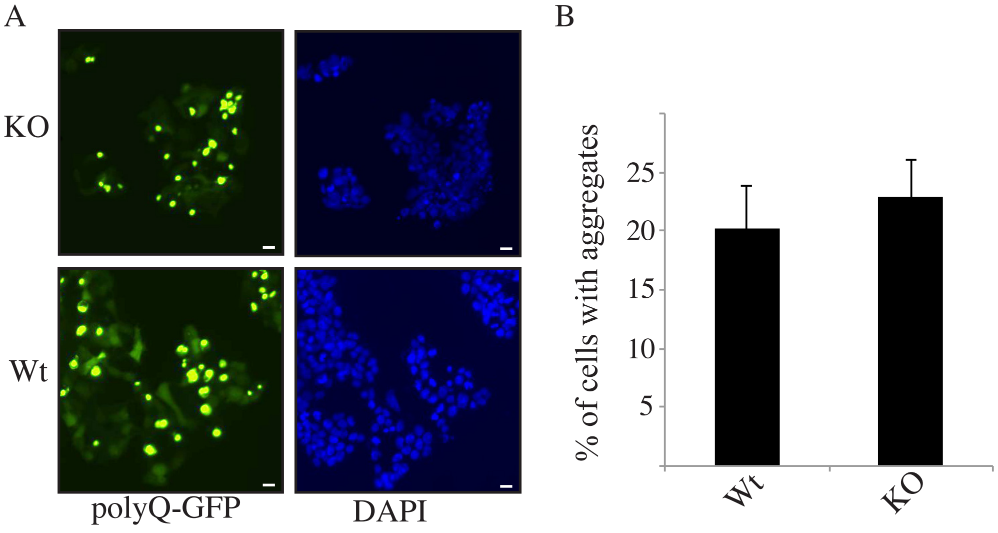

Supplementary figure 4: PolyQ aggregation is not suppressed by DNAJB6 in HEK293 cells
A, Images despicting Wt or DNAJB6 KO HEK293 cells transfected with PolyQ-GFP. B, quantification of the percentage of cells with PolyQ-GFP aggregates (n=3). Scalebar: 10 μM

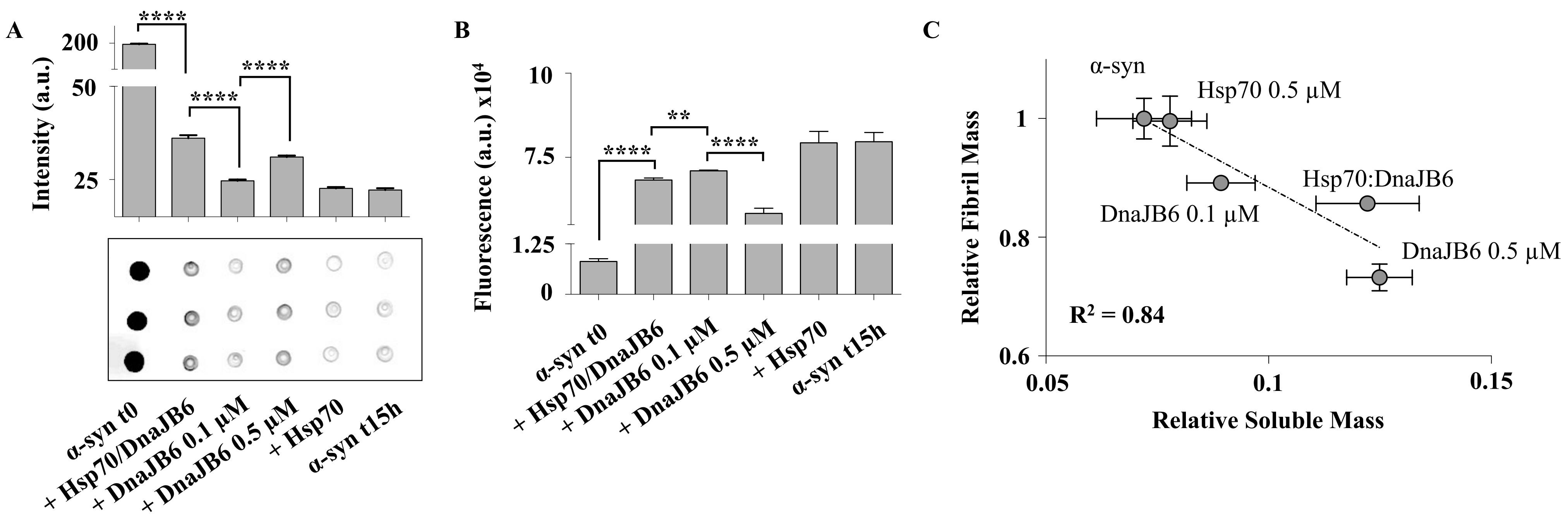

Figure S5. Validation of the ThT aggregation experiments. a) Dot blot experiments on α-syn aggregation mixtures in the absence or in the presence of different concentrations of Hsp70 and DnaJB6. Bottom, representative 3 replica- dot-blot experiment on soluble fractions of aggregation samples at 15 hours of incubation and, top, bar plot showing the densitometry analysis of the dot blots (n=9). **b)** ThT fluorescence values of α-syn aggregation mixtures in the absence or in the presence of different concentrations of Hsp70 and DnaJB6 at 15 h of incubation. c) Correlation between Relative fibril mass and relative soluble mass of α-syn aggregation mixtures at 15 h of incubation. Statistical analysis was performed by one-way ANOVA ($P<0.0001$) with post-multiple comparison (99% CI, * $P \leq 0.05$, ** $P \leq 0.01$, *** $P \leq 0.001$, **** $P \leq 0.0001$).

Supplementary Figure 6

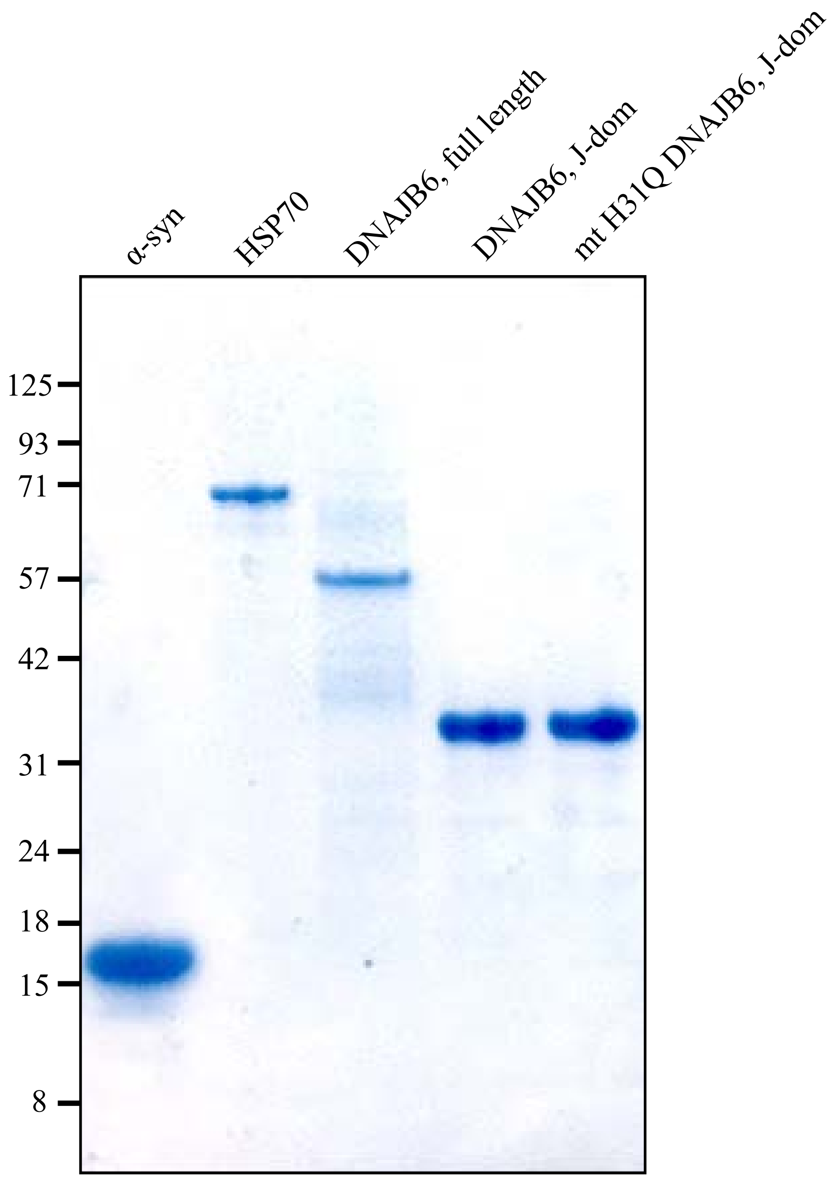

Supplementary Figure 6. Representative SDS-PAGE of the purified proteins used in this work. The DNAJ full length and J-domain proteins were produced as fusion proteins to GST.
